# The importance of a mathematical mindset in different academic disciplines

**DOI:** 10.3389/fpsyg.2026.1769464

**Published:** 2026-03-11

**Authors:** Xiaoyu Xu, Jo Boaler, Jack A. Dieckmann

**Affiliations:** 1 School of Mathematics, Guangdong University of Education, Guangzhou, Guangdong, China; 2 Graduate School of Education, Stanford University Graduate School of Education, Stanford, CA, United States

**Keywords:** educational intervention, gender, growth mindset, major, mathematical mindset

## Abstract

This study investigates how mathematical mindsets evolve in response to targeted pedagogical intervention, with a particular focus on disciplinary background. Drawing on data from a pre–post experimental design, we analyzed multiple dimensions of students’ mathematical mindset and achievement across distinct academic majors, also considering gender. While overall group-level changes were limited, dimension-specific improvements—particularly in attitudes toward mistakes and foundational skills—were observed. Engineering and Technology students consistently outperformed peers in all mindset dimensions, with the most pronounced gains in growth orientation and problem-solving strategies. Gender effects were comparatively minor and often embedded within disciplinary trends. Correlational and regression analyses revealed only a weak and gender-dependent link between mindset and achievement. These findings challenge the assumption of uniform mindset malleability and highlight the need for context-aware, discipline-sensitive interventions. Mathematical mindsets are not monolithic but structured and mediated by educational background, suggesting that future efforts to foster productive mindsets should be differentiated by academic context and target specific belief dimensions.

## 1 Introduction

Mathematical mindset, encompassing both cognitive beliefs about mathematical ability and emotional responses such as anxiety and resilience, plays a central role in shaping students’ mathematics learning experiences and academic performance (Boaler, 2016; Dweck, 2006; Hannula, 2006). Emerging from earlier growth mindset research that emphasized the malleability of ability through effort (Blackwell et al., 2007; Dweck, 2006), mathematical mindset extends this framework within the mathematics domain by incorporating students’ affective experiences and responses to challenge and error. While mindset-based interventions have sought to recalibrate students’ beliefs by normalizing mistakes and emphasizing learning processes (Boaler, 2016; Yeager et al., 2019), evidence in higher education—particularly in university-level mathematics—remains limited and mixed, suggesting that mathematical mindset at this stage is shaped not only by individual beliefs but also by structural features of the learning environment.

Among these structural features, gender and academic major have been identified as particularly salient. Although large-scale assessments indicate that overall mathematics achievement has become largely gender-neutral, qualitative differences persist in students’ confidence, anxiety, and interpretations of failure (OECD, 2019, 2023a). Female students are more likely to internalize difficulty as evidence of low ability, whereas male students more often attribute setbacks to external factors (Dweck, 2000; Eccles and Wigfield, 2002). These gendered patterns are further conditioned by disciplinary contexts. In non-STEM fields, where opportunities for sustained mathematical engagement are limited, students may develop more fragile or fixed orientations toward mathematics, whereas STEM environments often provide stronger institutional validation that may inadvertently reinforce gendered confidence gaps rather than resolve them (Nosek et al., 2009; Wang and Degol, 2013).

At the same time, research has increasingly emphasized that gender differences in mathematics-related beliefs and motivation are context-dependent rather than universal, varying across domains, educational stages, and institutional settings (Wang and Degol, 2016). Recent empirical work in China has further demonstrated that growth mindset is meaningfully associated with mathematics learning outcomes through affective and adaptive processes, underscoring the relevance of mindset-related constructs in exam-oriented educational systems (Chen et al., 2024). However, such studies have primarily focused on pre-tertiary populations and have rarely examined mathematical mindset as a multidimensional construct within higher education or in relation to disciplinary context.

Because disciplinary structures and gender norms are culturally embedded, findings derived largely from Western higher education systems may not readily generalize across contexts. China offers a particularly important case given its exam-oriented educational culture, large higher education population, and persistent gendered expectations surrounding mathematics (Cai, 2004; Leung, 2001). Building on scholarship that problematizes the treatment of mathematics learning as culturally “given,” particularly in STEM education, mathematical beliefs and practices in China are better understood as institutionally and socially shaped rather than as purely individual psychological traits (Li and Schoenfeld, 2019). In this context, mathematical mindset is more appropriately conceptualized as a socially situated and structurally shaped construct rather than a purely individual psychological trait, aligning with sociocultural perspectives that emphasize the co-construction of beliefs through interactions with peers, teachers, curricula, and broader cultural narratives (Esmonde, 2009; Nasir et al., 2008).

Against this theoretical backdrop, the present study examines how gender, academic major, and the cognitive and emotional subcomponents of mathematical mindset intersect in a non-Western higher education context. By adopting a multidimensional and context-sensitive perspective, this study seeks to clarify how structural and cultural factors shape students’ mathematical mindsets beyond achievement outcomes alone, while recognizing that the effectiveness of mindset interventions depends on broader social contexts, including gender norms, disciplinary cultures, and institutional practices (Walton and Cohen, 2011; Yeager and Walton, 2011).

Specifically, this study asks:

1. How do gender and academic major shape students’ mathematical mindsets in the context of Chinese higher education, where exam-driven culture and disciplinary stratification may intensify belief differentiation?
2. What dimensions of mathematical mindset—cognitive or emotional—exhibit the strongest gendered or disciplinary variation, and what patterns emerge when examining these subcomponents independently?
3. How might these insights contribute to a rethinking of equity in mathematics education, particularly by the expanding perspective from achievement outcomes to the underlying belief structures that support or hinder student engagement?

Through this analysis, we aim to challenge essentialist or deficit-oriented narratives about female students and to provide a nuanced account of how social identities and academic environments jointly structure students’ dispositions toward and experiences in mathematics. In doing so, the study contributes to a growing body of work that conceptualizes equity in education not just as equal performance, but as equal access to the development of positive learning dispositions and emotional resilience in mathematical contexts.

## 2 Literature review

### 2.1 Rethinking mathematical mindset in the context of gender and academic major

Building on prior work in educational psychology and mathematics education, mathematical mindset is increasingly understood as a domain-specific and multidimensional construct rather than a binary or unitary belief system. Extending earlier growth mindset research, this perspective emphasizes that students’ orientations toward mathematics encompass both cognitive dimensions—such as confidence in mathematical ability (MMH)—and emotional-regulatory dimensions, including anxiety management and responses to difficulty (MML) (Blackwell et al., 2007; Kim and Hodges, 2012).

In clarifying the conceptual scope of mathematical mindset, it is important to distinguish this construct from closely related beliefs such as self-efficacy or perceived competence. While self-efficacy typically refers to students’ judgments about their capability to perform specific mathematical tasks, mathematical mindset, as conceptualized in this study, captures a broader system of beliefs and affective orientations toward learning mathematics. This includes how students interpret difficulty, respond to errors, and regulate emotions during mathematical engagement, rather than evaluations of ability alone. In this sense, mathematical mindset is not reducible to confidence or competence beliefs, but reflects a domain-specific cognitive–emotional orientation toward mathematical learning processes and challenges.

Importantly, these dimensions are shaped not only by individual psychological processes but also by social and disciplinary contexts. Prior research indicates that gendered patterns in mathematical confidence and anxiety persist even when performance differences are minimal, suggesting that students’ mathematical mindsets are structured through broader cultural expectations rather than achievement alone (Goetz et al., 2013; OECD, 2023b; Rossi et al., 2022). Such patterns may be further conditioned by academic major. In non-STEM fields, where mathematics is often positioned as peripheral to disciplinary identity and institutional support for sustained engagement is limited, students may develop more fragile or fixed orientations toward mathematics than their peers in STEM contexts (Nosek et al., 2009; Wang and Degol, 2016).

From a sociocultural perspective, gendered beliefs about mathematical ability are reinforced through long-standing patterns of socialization and implicit messaging. Stereotypes that frame mathematics as a male domain, communicated by parents and teachers, have been shown to influence how students interpret struggle and failure, potentially contributing to the development of less adaptive mathematical mindsets among female students (Gunderson et al., 2017; Rattan et al., 2012). Rather than functioning as isolated psychological traits, mathematical mindsets thus emerge through interactions among individual beliefs, disciplinary norms, and institutional expectations.

Responding to calls for more context-sensitive and equity-oriented approaches to mindset research, particularly within non-Western higher education systems, the present study conceptualizes mathematical mindset as a socially situated, multidimensional construct. By disaggregating mindset into its cognitive and emotional components and examining their variation across gender and academic major, this study aims to clarify how structural and cultural factors shape students’ mathematical orientations beyond performance outcomes alone.

### 2.2 Gender, mindset, and the affective dimensions of mathematics learning

Despite progress in achieving gender parity in educational attainment, persistent differences remain in how male and female students perceive and engage with mathematics. Large-scale meta-analyses confirm that girls perform as well as boys in mathematical achievement (Else-Quest et al., 2010; OECD, 2023b), yet they consistently report lower confidence, higher anxiety, and reduced engagement (Goetz et al., 2013; Gunderson et al., 2012). These disparities are increasingly interpreted through the lens of gendered mathematical mindsets—that is, emotional and cognitive orientations toward mathematics shaped by gendered socialization and cultural expectations (Else-Quest et al., 2010).

Mathematical mindsets begin forming early in life and are shaped by the gendered messages children receive from parents, teachers, and society. Evidence that parents put into online searches “Is my son a genius?” 2.5 times more than “Is my daughter a genius?”—reveal implicit biases about intellectual potential and gender (Stephens-Davidowitz, 2014). These gendered beliefs in children’s potential may contribute to female students’ reduced sense of belonging in mathematics, especially in male-dominated academic environments (Dweck, 2008; Wang and Degol, 2016).

Although female students may show greater emotional regulation and awareness—traits associated with error tolerance and mindset resilience (Kim and Hodges, 2012)—these qualities often coexist with heightened sensitivity to failure and stereotype threat, both of which can suppress persistence and confidence in math learning (Cvencek et al., 2011). However, research also suggests that female college students are more likely to endorse growth mindsets in higher-level mathematics courses, and that these mindsets are positively associated with performance when reinforced by supportive pedagogical strategies (Olson, 2002).

Taken together, these findings suggest that achieving gender equity in mathematics involves more than narrowing performance gaps; it also requires addressing the gendered dynamics of mindset development and the messages given to girls and women that affect their achievement and participation in subjects. A deeper understanding of mathematical mindset as a socially constructed and affectively complex construct can inform more inclusive learning environments that support all students, particularly in culturally diverse and academically stratified settings.

### 2.3 Academic major and mathematical engagement

Academic major is another contextual factor that can be viewed as a contextual factor that interacts with students’ pre-existing beliefs—such as mathematics self-efficacy and identification—which have been shown to predict STEM major choice (Wang and Degol, 2013; Nosek et al., 2009). Cimpian et al. showed that professors in different fields varied in their belief that students needed a “gift” in order to succeed – a fixed ideas about innate ability. The more professors believed that students needed “a gift” the fewer women and minority students were in that field (Leslie et al., 2015). Students who choose STEM fields often report higher mathematics self-efficacy and stronger identification with mathematical practices – probably because they have had to develop these traits to stay inside STEM. At the same time, the institutional and cultural features of STEM majors may further reinforce or reshape particular mindset subcomponents, such as emotional resilience or attitudes from failure. Some research has found that non-STEM students are more likely to disengage from mathematics and adopt fixed mindsets, but findings are inconsistent (Hannula, 2002).

### 2.4 Gender, major, and the cultural context of belief development

Few studies have examined how gender and academic major jointly shape mathematical beliefs in Eastern contexts. In China, high academic pressure, exam-oriented instruction, and gender role expectations may intensify belief differentiation (Cai, 2004; Leung, 2001). Female students in non-STEM majors may face both internalized stereotypes and reduced institutional support for mathematics, leading to distinct emotional-motivational profiles compared to their male or STEM counterparts. Understanding gendered belief structures in this cultural context is crucial to addressing equity and inclusion in mathematics education.

### 2.5 Summary and conceptual framework

**Figure 1:**
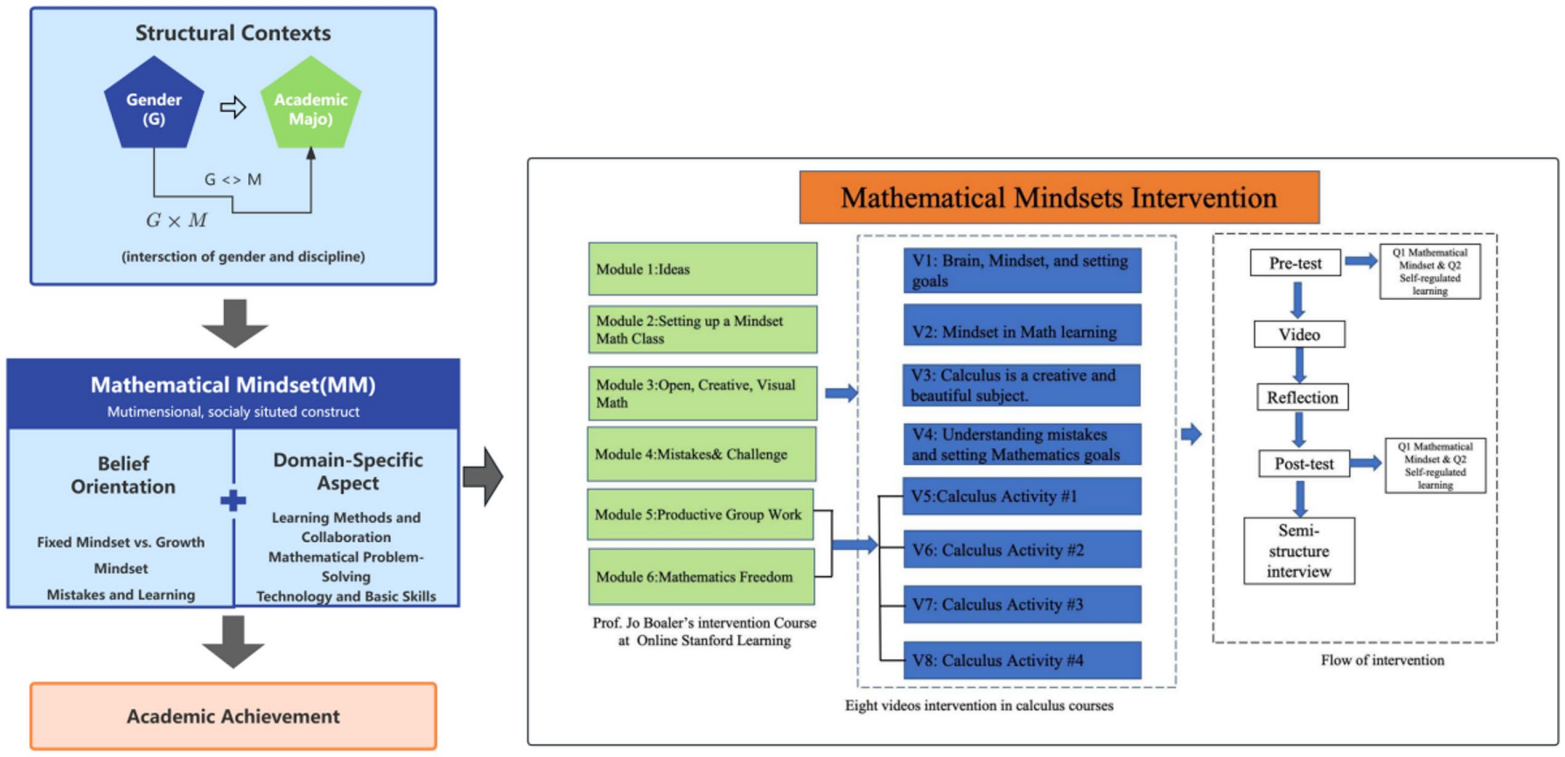
Conceptual framework of the present study and its connection to the prior mathematical mindset intervention. The left panel illustrates the conceptual framework examined in the present study, focusing on how structural contexts (gender and academic major) relate to multidimensional mathematical mindset and academic achievement. The right panel summarizes the mathematical mindset intervention implemented in our prior research (Xu et al., 2025), which provides the empirical basis for the current analyses. The intervention framework is adapted from Xu et al. (2025) and is included here to clarify the analytical context rather than to introduce a new intervention.

In sum, prior studies have not adequately addressed how gender and academic major shape mathematical mindset as a multidimensional construct within higher education contexts. This study contributes to the literature by (1) disaggregating mathematical mindset into its cognitive and emotional components, (2) examining how these components vary across gender and academic major, and (3) interpreting these patterns within the sociocultural context of Chinese higher education. As illustrated in the conceptual framework (see Figure 1), gender and academic major are theorized as structural contexts that condition students’ experiences with mathematics, shaping multidimensional mindset orientations that are, in turn, associated with academic achievement. The framework also situates the present analysis in relation to the previously implemented mathematical mindset intervention, clarifying how the current study builds on earlier work while shifting analytical focus from intervention design to structural variation in post-intervention outcomes.

## 3 Methodology

This study employed a cross-sectional design, drawing on the research framework and dataset established in our previous studies (Xu and Dieckmann, 2025; Xu et al., 2025). In January 2023, the intervention group participated in a two-week online video–based mathematical mindset program adapted from Jo Boaler’s Stanford Online Learning course; in the present study, this intervention serves as a shared instructional background rather than the focus of effectiveness evaluation.

While the research design and participant characteristics remained consistent with the previous studies, the present investigation extends the analytical focus by examining how gender and academic major shape variation across the five dimensions of mathematical mindset, as well as their subsequent associations with mathematics achievement. All statistical analyses for this analytical extension were conducted using RStudio, with the aim of providing a more nuanced understanding of how individual differences in gender and academic major interact with students’ mindset dimensions in higher education mathematics contexts.

### 3.1 Participants and data collection

Data were collected through an online questionnaire, which included a Mathematical Mindset Questionnaire (MMQ) assessing five key dimensions of mathematical mindset: Fixed Mindset vs. Growth Mindset, Mistakes and Learning, Learning Methods and Collaboration, Mathematical Problem-Solving, and Technology and Basic Skills. The Technology and Basic Skills dimension specifically focused on students’ ability to apply mathematical tools, such as calculators and mathematical software, and their mastery of foundational skills essential for advanced problem-solving. Mathematics achievement was assessed using a standardized test, with scores ranging from 0 to 20. The study, conducted at a university in Guangdong, China, initially considered 1,334 students eligible for participation, but 892 were excluded for reasons such as opting out or issues with pre-test data (e.g., incomplete or contradictory responses). Consequently, 442 students, stratified by gender and representing a diverse range of academic disciplines, including technology, engineering, art, business, and English, were included in the final analysis (Xu et al., 2025).

### 3.2 Analytical extensions

This study extends previous analyses by exploring the influence of gender and academic major on mathematical mindset, providing a nuanced understanding of how these demographic factors shape students’ mindset orientations. Furthermore, the study offers an in-depth examination of the five mathematical mindset dimensions, focusing on the ways these dimensions evolve and their relationship with mathematics achievement. Finally, multivariate analysis is employed to assess the combined effects of these mindset dimensions on academic performance, facilitating a comprehensive understanding of the interactions between mindset factors and their impact on student outcomes. To clarify how these dimensions were operationalized in the present study, Table 1 provides an overview of the five dimensions of the Mathematical Mindset Scale along with their corresponding sample item numbers. These dimensions capture various aspects of students’ mathematical mindset, including their beliefs about effort, learning from mistakes, collaboration strategies, and the use of technology and basic mathematical skills.

**Table 1:** Dimensions of the mathematical mindset scale.

Component	Dimension	Sample Item No(s).
Belief orientation	Fixed mindset vs. Growth mindset	Q1, Q5, Q11, Q14
	Mistakes and learning	Q2, Q7
Domain-specific aspect	Learning methods and collaboration	Q4, Q8, Q10
	Mathematical problem-solving	Q3, Q6, Q13
	Technology and basic skills	Q15, Q17

GM = Fixed Mindset vs. Growth Mindset; LMS = Learning Mindset Scale; ML = Mistake Learning; PLS = Mathematical Problem-Solving; TBS = Technology and Basic Skills. The category “Fixed Mindset vs. Growth Mindset” includes GM, LMS, ML, PLS, and TBS, and is grouped based on thematic alignment of survey items. In this study, fixed versus growth mindset represents a general belief orientation toward mathematical ability, whereas the remaining dimensions capture domain-specific cognitive, affective, and behavioral manifestations of mathematical mindset in learning contexts.

The mathematical mindset scale used in this study was developed to assess students’ beliefs and attitudes toward mathematics learning across five distinct dimensions. These dimensions were derived based on thematic grouping of 17 survey items and were aligned with the broader theoretical construct of fixed versus growth mathematical mindset. The first dimension, Fixed Mindset vs. Growth Mindset (GM), includes Items Q1, Q5, Q11, and Q14, and captures students’ core beliefs about whether mathematical ability is innate or malleable through effort and persistence. The second dimension, Mistakes and Learning (ML), represented by Items Q2 and Q7, evaluates the extent to which students view errors as valuable learning opportunities. The third dimension, Learning Methods and Collaboration (LMS), comprising Items Q4, Q8, and Q10, assesses students’ openness to adopting various strategies and engaging in collaborative learning. The fourth dimension, Mathmatical Problem-Solving (PLS), consisting of Items Q3, Q6, and Q13, reflects attitudes toward balancing algorithmic procedures with flexible problem-solving approaches. Finally, the Technology and Basic Skills (TBS) dimension, captured by Items Q15 and Q17, measures students’ beliefs about the role of technological tools in relation to fundamental mathematical skills. While each dimension represents a specific aspect of mathematical mindset, together they collectively reflect students’ orientation toward learning mathematics and are conceptually nested under the overarching construct of growth-oriented beliefs.

### 3.3 Statistical approach: two-way ANOVA for gender and major effect

To ensure that subsequent comparisons across gender and academic major were grounded in a psychometrically defensible measurement structure, we first examined the factorial suitability of the mathematical mindset items. An exploratory factor analysis (EFA) was conducted on the pre-test data using maximum likelihood extraction with oblimin rotation, reflecting the theoretical expectation that mindset dimensions are correlated. The Kaiser–Meyer–Olkin (KMO) measure indicated acceptable sampling adequacy (KMO = 0.65), and Bartlett’s test of sphericity confirmed that the correlation matrix was factorable [χ^2^(105) = 704.17, *p* < 0.001]. The resulting five-factor solution accounted for 43.6% of the total variance, providing preliminary overall evidence in support of the intended multidimensional structure of mathematical mindset. Although two dimensions (Mistakes and Learning; Technology and Basic Skills) were represented by only two items, they were retained to preserve conceptual coverage of theoretically meaningful aspects of the construct; accordingly, interpretations emphasize patterns of group differences rather than reliance on subscale-specific internal consistency alone. Internal consistency estimates (Cronbach’s *α*) for all subscales are reported in Table A1 for completeness; however, given the exploratory nature of the study, the conceptually heterogeneous structure of mathematical mindset, and the limited number of items in several subscales, these coefficients were treated as supplementary information rather than as the primary basis for inference.

Building on this measurement foundation, a series of two-way ANOVAs were conducted to examine the effects of gender, academic major, and their interaction on students’ post-intervention mathematical mindset scores (see Table 2). The results revealed a consistent pattern across all five mindset dimensions and the total score, with academic major emerging as the most influential factor. Major significantly affected all outcomes (*p* < 0.05), with effect sizes ranging from small to very large. Specifically, major had a large effect on Post-Growth Mindset (η^2^ = 0.52), Post-Mathematical Problem Solving (η^2^ = 0.23), and Post-Technology and Basic Skills (η^2^ = 0.25), and an exceptionally large effect on the total mindset score (η^2^ = 0.86), indicating that students from different academic disciplines responded to the intervention in notably different ways. Although gender only had a mildly significant effect on individual mindset measures (*p* < 0.1 level) it showed a significant main effect on the total mindset score, *F*(1, 108) = 13.22, *p* < 0.001, η^2^ = 0.11, indicating a moderate gender-based difference in overall mindset. However, there were no significant interaction effects between gender and major for any of the variables, and all corresponding effect sizes were negligible (η^2^ ≤ 0.05), indicating that the influence of academic major on mathematical mindset was consistent across gender groups. Taken together, these findings suggest that gender played a role in shaping students’ overall mindset in this study, but academic major exerted the strongest and most consistent influence on post-intervention outcomes.

**Table 2:** Two-way ANOVA results for post-intervention mathematical mindset scores by gender and major.

Outcome	Effect	F(df1, df2)	*p*-value	partial η^2^
PoGM	Gender	0.11 (1, 108)	0.744	<0.001
	Major	39.41 (3, 108)	<0.001 ***	0.52
	Gender × Major	0.85 (3, 108)	0.471	0.02
PoML	Gender	0.02 (1, 108)	0.897	<0.001
	Major	5.73 (3, 108)	0.001 **	0.14
	Gender × Major	0.70 (3, 108)	0.556	0.02
PoLMS	Gender	0.01 (1, 108)	0.933	<0.001
	Major	2.79 (3, 108)	0.044 *	0.07
	Gender × Major	0.38 (3, 108)	0.770	0.01
PoPLS	Gender	0.01 (1, 108)	0.905	<0.001
	Major	10.55 (3, 108)	<0.001 ***	0.23
	Gender × Major	0.48 (3, 108)	0.700	0.01
PoTBS	Gender	0.13 (1, 108)	0.716	<0.001
	Major	11.84 (3, 108)	<0.001	0.25
	Gender × Major	0.89 (3, 108)	0.447	0.02

**Asterisks indicate statistical significance: *p < .05, **p < .01, and ***p < .001.

## 4 Results

### 4.1 Baseline effects of the intervention on mathematical mindsets

**Figure 2:**
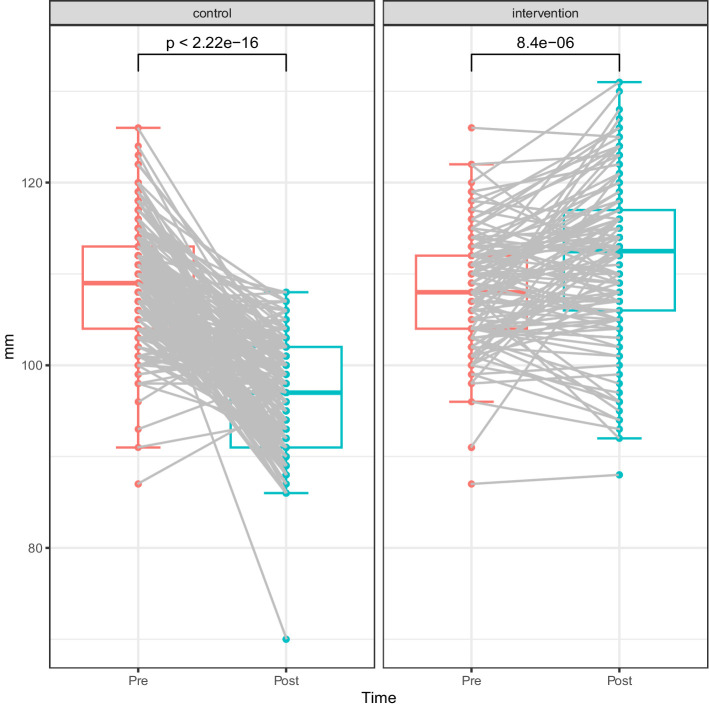
Mathematical mindset trajectories: Control vs. intervention.

To evaluate, a comparison of pre- and post-intervention scores for the Control and Intervention groups reveals distinct patterns of change, with each line representing an individual participant’s score shift from pre- to post-intervention (Figure 2), highlighting the statistical significance of the observed effects. To further understand the impact, let us first examine the changes in mathematical mindset brought about by this intervention. The *x*-axis shows the time points (pre and post), while the y-axis represents the scores for the intervention measures. Based on the data and visualizations, the plot compares the changes in scores (measured as “mm”) for both the Control and Intervention groups, focusing on pre- and post-intervention outcomes. The control group exhibits a relatively narrow distribution of scores, showing a modest decline from pre- to post-intervention. This decline is reflected by the smaller spread of data points and the lack of substantial changes across participants. On the other hand, the intervention group shows a wider distribution, with several participants experiencing a noticeable increase in scores from pre- to post-intervention. This greater variability is reflected in the larger spread of scores and the presence of more outliers, particularly in the post-intervention phase.

Statistically, the Control group has a *p*-value of 2.22e-16, indicating a highly significant change in scores, suggesting a decline or no effective intervention impact. In contrast, the Intervention group has a p-value of 8.4e-06, which reflects a statistically significant improvement, supporting the effectiveness of the intervention. These findings suggest that while the Control group saw little improvement, the Intervention group showed a positive change in scores. While the observed effect on total mathematical mindset is of moderate magnitude, it remains practically meaningful in the context of educational research, particularly given the exploratory nature of this analysis and the brief intervention period. Such moderate effects may reflect meaningful shifts in students’ mathematical beliefs and dispositions, which are expected to influence longer-term engagement rather than immediate, large-scale gains. However, the wider spread in the Intervention group indicates considerable variability in individual responses to the intervention. This variability highlights the need for further investigation to understand the factors influencing individual changes and to optimize strategies for enhancing the intervention’s effectiveness across diverse student populations.

### 4.2 Variation in mathematical mindsets by gender and academic major

**Figure 3:**
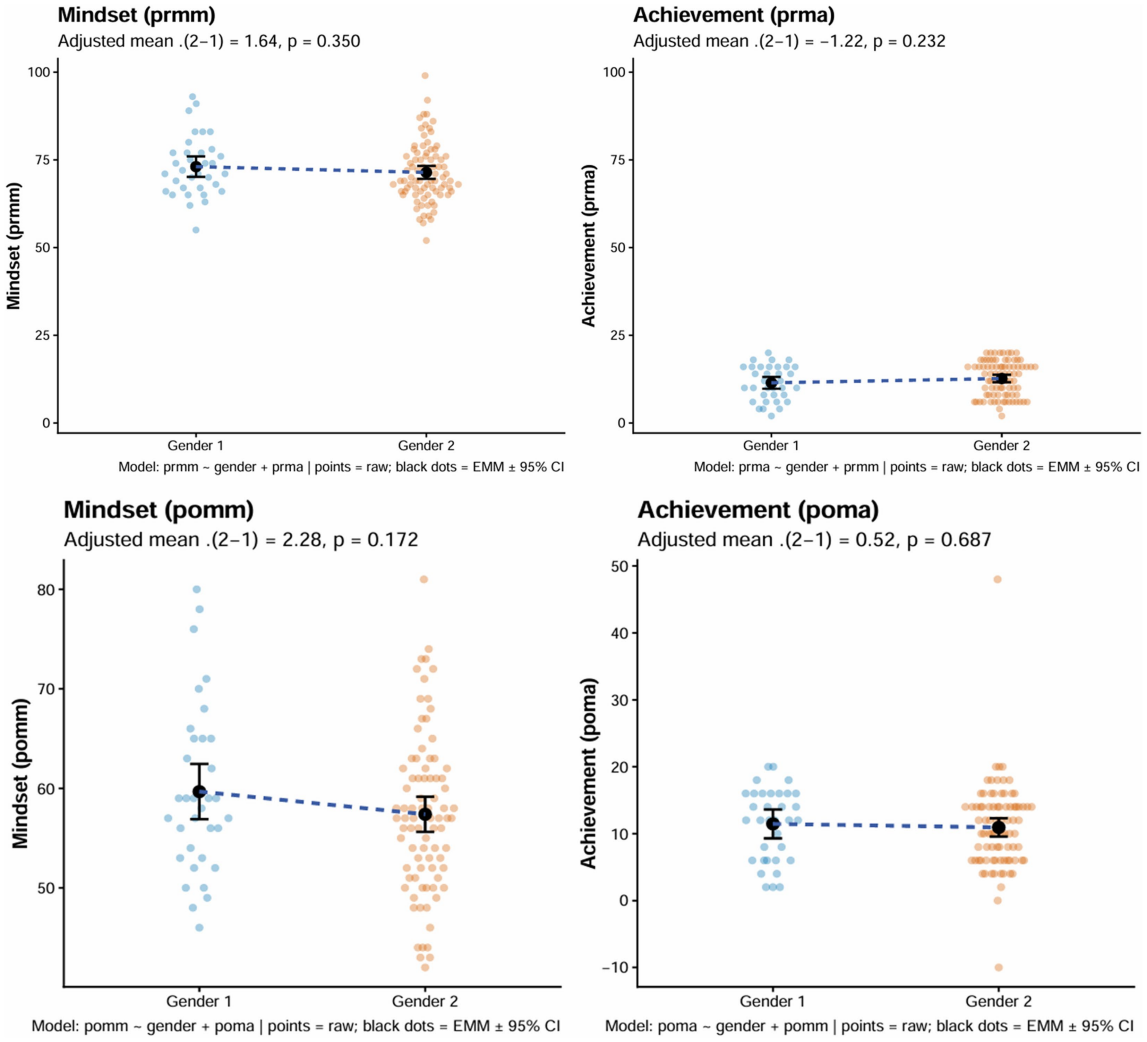
Gender differences in pre- and post-test mathematical mindset and achievement scores.

To examine the relationship between gender and the outcomes depicted in Figure 3, adjusted female–male contrasts were calculated using linear models estimating Estimated Marginal Means (EMMs) by gender (Male = 1, Female = 2), while conditioning on the complementary construct in each panel. The plot elements are as follows: the light-colored scatter points represent individual raw observations (with blue denoting Gender 1 (Male) and orange representing Gender 2 (Female)), while the black dots indicate the adjusted EMMs for each gender, accompanied by black error bars showing the corresponding 95% confidence intervals (CIs). The dashed lines connecting the adjusted means for both genders visually represent the female–male contrast (*Δ*(f–m)), where the slope of the dashed line indicates the direction and magnitude of the gender difference: an upward slope (from Gender 1 to Gender 2) suggests that the female EMM is higher than the male EMM (*Δ* > 0), while a downward slope implies the opposite (*Δ* < 0). A nearly horizontal dashed line suggests no meaningful gender difference (Δ ≈ 0). Taken together, these elements allow Figure 2 to display the joint distribution of gender with pre- and post-test mindset and achievement.

The scatter plots accompanying these analyses show that the point clouds for Gender 1 (Male) and Gender 2 (Female) overlap extensively in all four panels and that within-gender variability is pronounced and generally exceeds the between-gender differences. Specifically, prmm and prma (pre-test mindset and achievement) exhibited more concentrated scores for Gender 1 (Male), with most observations for both genders clustering in the mid-range of the scale but some spread toward lower and higher values, while both pomm and poma (post-test mindset and achievement) demonstrated greater dispersion across both genders, with Gender 1 (Male) showing a wider spread of data points that fan out over a larger portion of the 0–100 scale. Consistent with these visual patterns, the adjusted contrasts for the four outcomes were consistently small and statistically non-significant: for prmm (Pre-test Mindset), Δ = +1.64, *p* = 0.350, with a slight upward slope; for prma (Pre-test Achievement), Δ = −1.22, *p* = 0.232, showing a slight downward slope; for pomm (Post-test Mindset), Δ = +2.28, *p* = 0.172, with a slight upward slope; and for poma (Post-test Achievement), Δ = +0.52, *p* = 0.687, exhibiting a nearly horizontal line. In all cases, the 95% confidence intervals for these contrasts included zero, indicating no significant gender differences. Furthermore, the absolute magnitude of the differences was less than 3 points on a 0–100 scale, suggesting negligible practical significance.

Taken together, these dispersion patterns indicate that the observable shifts in post-test data are better interpreted as individual variability within each gender than as evidence of a systematic gender effect. Therefore, based on the adjusted analyses, the black dots (EMMs) and the near-horizontal dashed lines, coupled with the overlapping confidence intervals, reinforce the conclusion that any gender differences in mindset and achievement are small and statistically non-significant. The results suggest that, after adjusting for the complementary construct, gender does not play a substantial role in determining the outcomes of mindset and achievement in this study.

**Figure 4:**
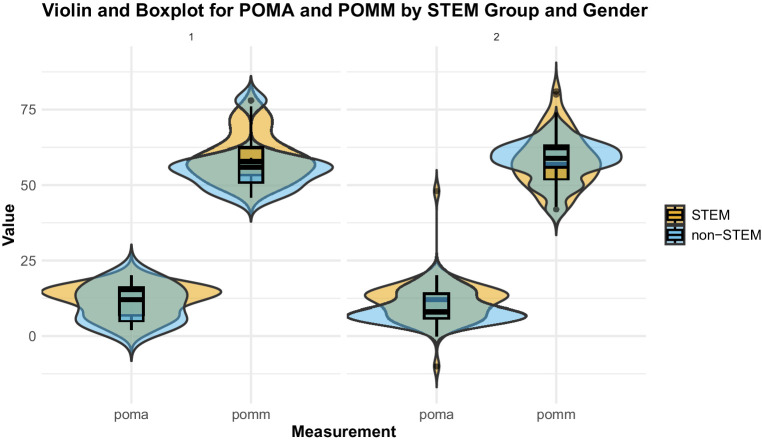
Comparison of post-test mathematical mindsets and achievement by major group (STEM vs. non-STEM) and gender.

To examine the combined influence of gender and disciplinary background, we constructed violin plots comparing post-test scores for mathematical mindset and achievement across STEM and Non-STEM groups (Figure 4). For the purposes of this analysis, majors were collapsed into STEM (Engineering and Technology) and Non-STEM (Business and Art), allowing clearer comparisons across disciplinary domains. It displays post-test outcomes for pomm (post-test mindset) and poma (post-test achievement) within gender facets (1 = male, 2 = female), using overlaid violin density plots and boxplots (*x*-axis = measure; *y*-axis = score; orange = STEM, blue = non-STEM). Violins represent kernel-density estimates of the full distribution, while the embedded boxplots summarize the median (thick line), interquartile range (IQR) (box), non-outlying range (whiskers), and outliers (points beyond whiskers). Outliers are defined as data points that fall outside the non-outlying range, represented as individual points beyond the whiskers, indicating extreme values that deviate significantly from the central distribution. STEM students exhibited overall higher values than their Non-STEM peers. Within STEM, females demonstrated slightly elevated medians and reduced variability, suggesting more consistently positive orientations toward mathematics compared to males. This likely reflects the finding that it is girls who have developed a growth mindset who persist in STEM majors (Dweck, 2006).

Two key distributional patterns emerge. Central tendency: within each gender facet, STEM and non-STEM medians are closely aligned for both pomm and poma; any separation is modest, with a slight elevation in the STEM group, most visible in poma. Dispersion and shape: STEM groups exhibit consistently larger IQRs, longer whiskers, broader violins, and heavier upper (occasionally lower) tails, indicating greater within-group heterogeneity and a higher incidence of extreme values. By contrast, non-STEM distributions are narrower and more centrally concentrated, with fewer outliers. The variance contrast is most pronounced for poma and remains evident for pomm. Conditional on STEM status, male and female distributions show similar medians and distributional shapes, implying limited gender separation in central tendency; the key distinction is a systematically higher variance among STEM participants.

Collectively, the figure suggests that STEM status is associated primarily with dispersion rather than with a mean shift in post-test mindset or achievement. The presence of more outliers in STEM groups, particularly in poma, further underscores the greater variability in STEM students’ mindset and achievement outcomes. These findings highlight the importance of variance-aware, differentiated support strategies, which are more relevant than interventions based solely on average differences.

### 4.3 Dimensional patterns of gendered and disciplinary differences in mindset

**Figure 5:**
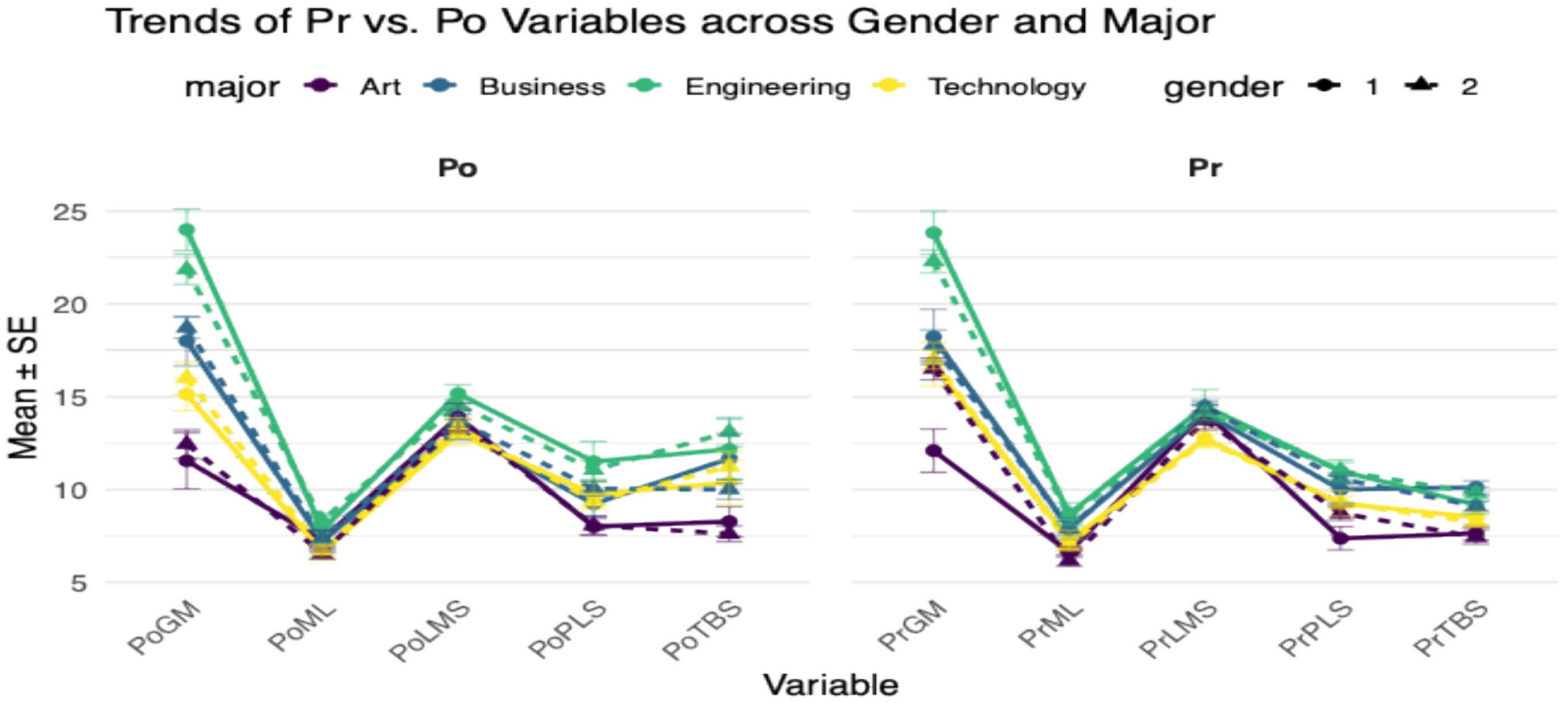
Pre–post trends in mathematical mindset dimensions across gender and academic major.

Substantial variation was observed across mathematical mindset dimensions when scores were disaggregated by gender and academic major. Across ten assessed variables—five pre-intervention (PrGM, PrML, PrLMS, PrPLS, PrTBS) and five post-intervention (PoGM, PoML, PoLMS, PoPLS, PoTBS)—students exhibited distinct profiles aligned with their disciplinary backgrounds. Each dimension captures a different facet of students’ beliefs and behaviors related to mathematics, ranging from growth orientation and learning from mistakes to collaboration, problem-solving strategies, and basic skills (see Figure 5).

Visual comparison of mean scores, grouped by gender (coded as 1 = male, 2 = female) and color-coded by major—Art (purple), Business (blue), Engineering (green), and Technology (yellow)—reveals clear differences (See Figure 4). Students in Engineering consistently demonstrated the highest scores across most dimensions, particularly in Growth Mindset (GM), Problem-Solving Strategies (PLS), and Learning Methods and Collaboration (LMS), suggesting a stronger alignment with productive learning beliefs. Business and Technology majors followed with moderate scores, while Art majors generally scored lowest across both pre- and post-intervention phases. These patterns remained relatively stable before and after the intervention, though small post-intervention gains were observed in certain subgroups.

Gender-based differences, while present, were less pronounced than disciplinary effects. Most gender pairs within the same major exhibited overlapping standard errors, indicating minimal divergence between male and female students. However, in select cases (e.g., PoGM, PoPLS), slight differences emerged, with female students in some majors outperforming their male peers. Taken together, these findings suggest that academic major functions as a more influential factor than gender in shaping mathematical mindset dimensions at both pre- and post-intervention stages.

Building upon the group-level differences observed in mean scores across mindset dimensions (see Figure 5), further analysis was conducted to examine how these dimensions evolved from pre- to post-intervention across gender and academic major groups. Figure 4 displays line plots comparing the mean scores and standard errors of five core dimensions—Growth Mindset (GM), Mistakes and Learning (ML), Learning Methods and Collaboration (LMS), Problem-Solving Strategies (PLS), and Technology and Basic Skills (TBS)—before (Pr) and after (Po) the intervention. Each line represents a subgroup defined by gender (solid = male, dashed = female) and academic major (color-coded as Art, Business, Engineering, or Technology), allowing for the visualization of longitudinal trends.

**Figure 6:**
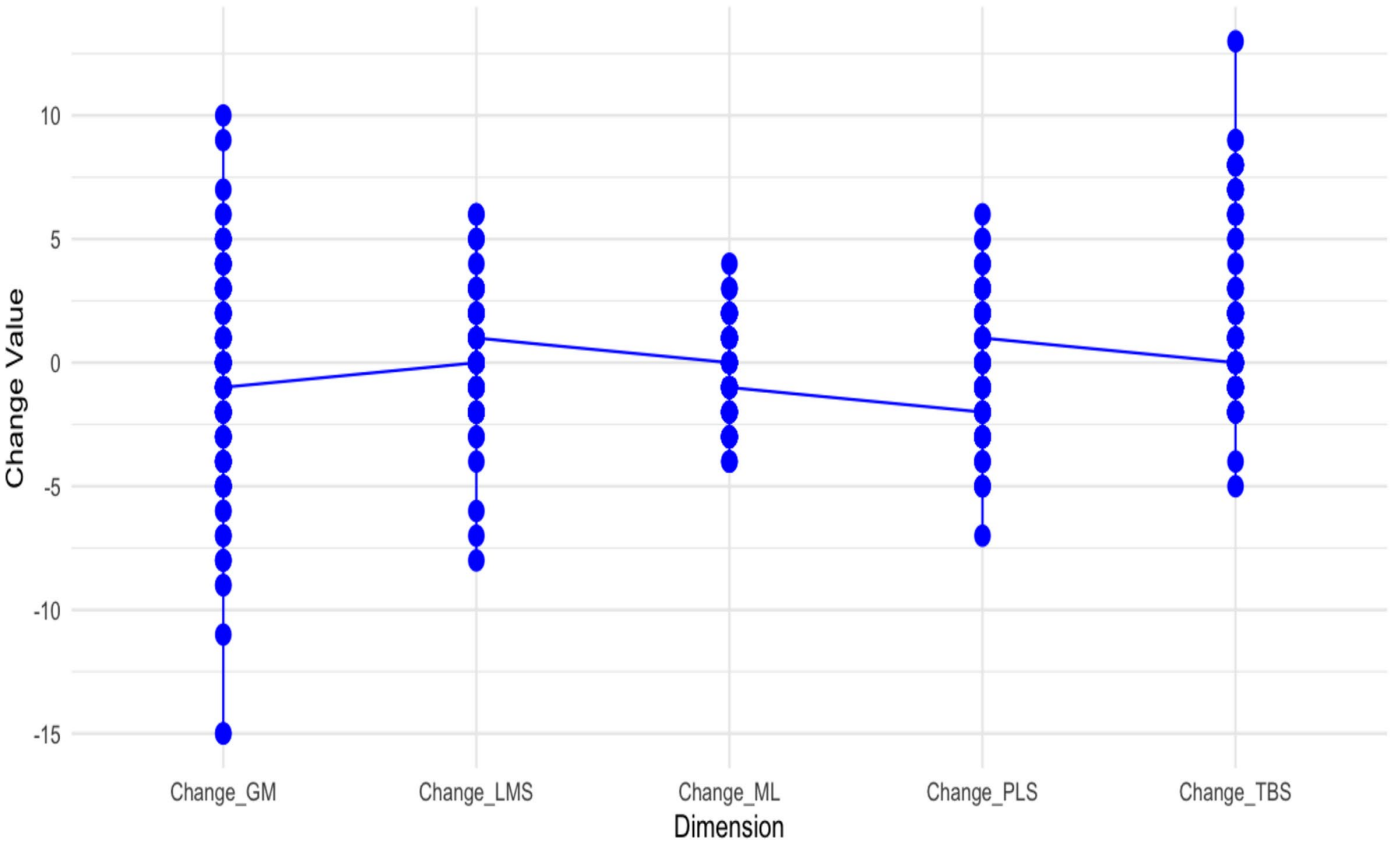
Changes in mathematical mindset dimensions.

Consistent with the patterns observed in Figure 6, Engineering majors demonstrated the highest overall scores at both time points, particularly in GM and PLS, suggesting stronger baseline orientations and greater receptivity to growth-based learning in the context of mathematics. Business and Technology students followed with intermediate scores, while Art majors again exhibited the lowest averages, especially on GM and PLS. The trajectories of Po scores largely mirror their Pr profiles, although slight post-intervention increases are visible in select variables (e.g., PoLMS, PoTBS), particularly among Engineering and Technology students—indicating possible intervention effects in domains related to collaborative learning and technical skill confidence.

Gender-related differences remain minimal across all majors, as reflected in the small vertical gaps between solid and dashed lines. Nevertheless, subtle variations emerge in a few dimensions, such as higher GM and PLS scores for female students in Engineering and Business. While not statistically tested in this figure, these trends may suggest gender-by-major interactions worth further exploration. Taken together, the line plots reinforce the role of academic discipline as the primary differentiating factor in students’ mathematical mindsets, with gender differences being relatively modest and secondary.

While the previous visualizations delineated cross-sectional group differences and longitudinal trajectories in mindset scores by major and gender (see Figures 5, 6), a further layer of insight can be gained by examining individual-level change patterns. To synthesize both individual and aggregated dynamics, Figure 6 presents the distribution and mean change scores across five dimensions of mathematical mindset, calculated as the difference between post- and pre-intervention scores for each participant.

The figure reveals that the most substantial positive shifts occurred in Growth Mindset (Change_GM) and Technology and Basic Skills (Change_TBS), suggesting that students developed stronger beliefs in the malleability of mathematical ability and became more appreciative of technological tools and foundational competencies in learning mathematics. Moderate but consistent improvements were also evident in Mistakes and Learning (Change_ML) and Learning Methods and Collaboration (Change_LMS), implying increased receptivity to learning from errors and collaborative engagement. In contrast, the Mathematical Problem-Solving (Change_PLS) dimension showed a slight overall decline, indicating that some students may have continued to associate mathematics with rigid procedural tasks rather than flexible reasoning.

In aggregate, the intervention preferentially strengthened growth-oriented beliefs and technology-related competencies, while procedural epistemologies exhibited limited malleability over the study interval. These patterns underscore the importance of designing targeted instructional scaffolds—such as reflective error analysis, exploratory problem-solving contexts, and structured peer collaboration—to facilitate more balanced and transformative shifts in students’ mathematical mindsets.

**Figure 7:**
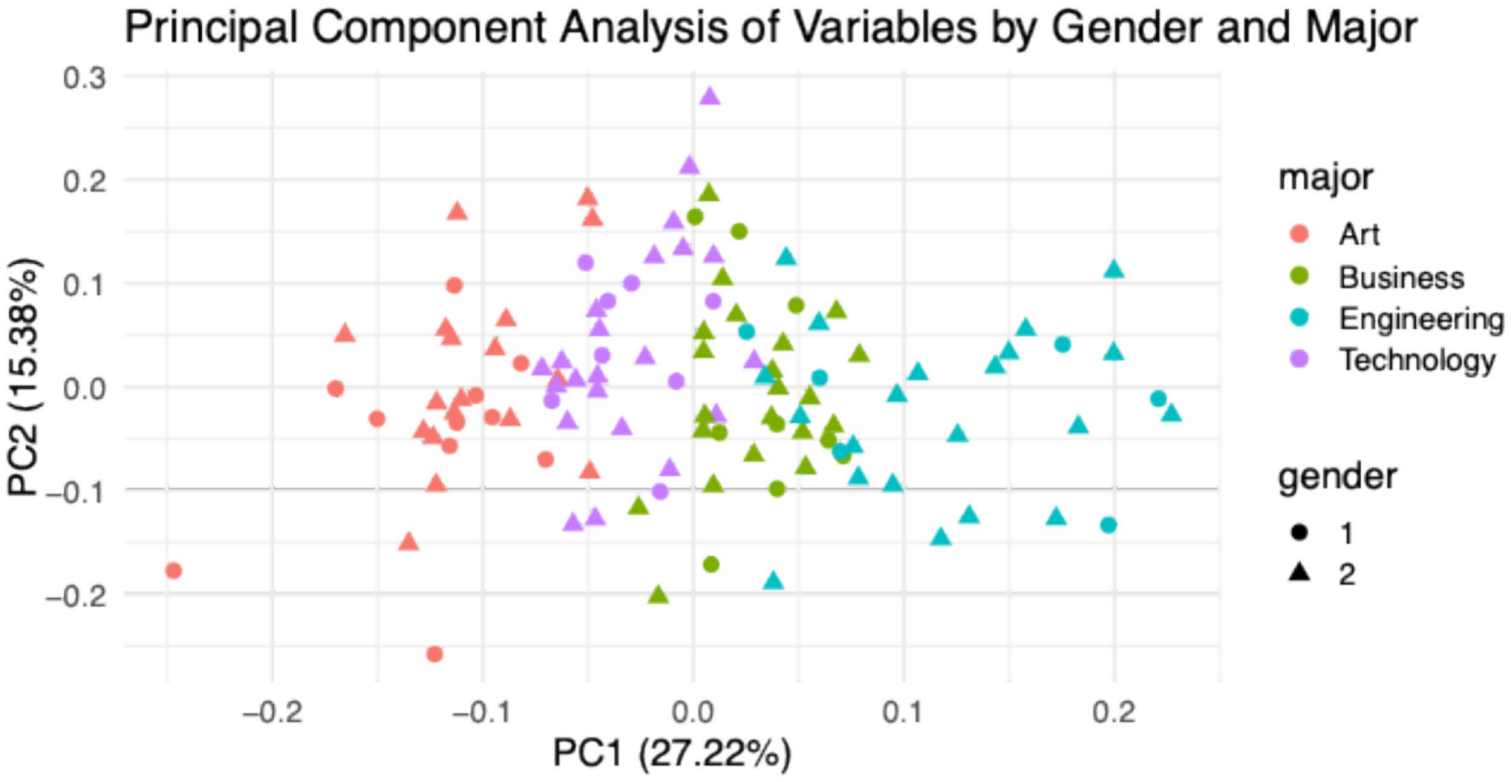
Principal component analysis of mathematical mindset profiles by major and gender.

To further examine the multivariate structure underlying students’ mindset profiles, Figure 7 presents a two-dimensional Principal Component Analysis (PCA) biplot derived from the 10 mindset-related variables assessed in the study. The first two principal components—PC1 (27.22%) and PC2 (15.38%)—together explain 42.6% of the total variance in the dataset. Each point in the plot represents an individual student, with color denoting academic major (Art = red, Business = green, Engineering = cyan, Technology = purple) and shape indicating gender (circle = male, triangle = female). This dimensionality-reduction approach allows for the visualization of latent psychological clusters and patterns that are not easily captured by univariate comparisons.

Clear disciplinary clustering is observed, most notably among Engineering and Art students. Engineering majors (cyan) are distributed predominantly along the positive end of PC1, indicating strong loadings on latent dimensions associated with high mindset scores, such as growth orientation and flexible problem-solving strategies. In contrast, Art students (red) cluster toward the negative end of PC1, reflecting comparatively lower overall endorsement of these constructsin context of mathematics learning. Business (green) and Technology (purple) students occupy more central and overlapping regions of the plot, suggesting either shared cognitive-motivational patterns or greater within-group heterogeneity in those domains.

In contrast, gender-related differentiation in PCA space appears minimal. Male (circles) and female (triangles) students from the same major generally overlap in their distribution, indicating that academic major, rather than gender, is the dominant source of variance in the underlying mindset dimensions. Slight vertical spread along PC2 may reflect nuanced within-major gender variation in secondary traits—such as perceptions of mistakes or collaboration—but these differences are subtle and non-clustered. Overall, the PCA results reinforce earlier findings by illustrating discipline-specific psychological profiles in mathematical mindset development, underscoring the critical role of academic context in shaping students’ beliefs, motivations, and cognitive approaches toward learning mathematics.

## 5 Discussion

### 5.1 Clarifying mindset: disciplinary context, gender nuance

The results reveal a consistent pattern across analyses: within this sample, variation in mathematical mindset in this sample is more strongly associated with disciplinary context than with gender (Figure 2). At the cohort level, gender differences were small, and within STEM fields female students exhibited slightly higher and more stable mindset scores on selected dimensions. Rather than indicating broad gender-based divergence, these patterns suggest that disciplinary affiliation introduces meaningful within-group heterogeneity while leaving overall gender-based means largely comparable. This finding aligns with prior research indicating that achievement-related beliefs are malleable yet context-dependent (Dweck, 2006; Yeager and Dweck, 2012), that gender differences in such beliefs tend to be modest at the aggregate level (Hyde, 2005), and that disciplinary environments play an important role in shaping motivational and cognitive outcomes (Wang and Degol, 2016).

Disaggregating mathematical mindset into five dimensions further reinforces this conclusion (Figure 6). Engineering—and, to a lesser extent, Technology/Business— showed higher mean scores across several dimensions, whereas Art consistently showed lower scores, particularly in Growth Mindset and Problem-Solving Strategies. Changes over time also varied by field: STEM majors demonstrated modest, dimension-specific gains, while non-STEM majors exhibited flatter or more heterogeneous trajectories. Gender differences across dimensions were intermittent and small, occasionally favoring female students in Growth Mindset and Problem-Solving, but no uniform gender pattern emerged across dimensions.

Consistently, the PCA results presented in Figure 7 indicate that academic major, rather than gender, accounts for the primary structure of variance in students’ mathematical mindset profiles. The analysis reveals clear discipline-based clustering, with students in Engineering and Art occupying relatively distinct regions of the component space, while gender-related variation appeared largely overlapping. This pattern is consistent with the view that disciplinary “cultures” organize motivational affordances (Leslie et al., 2015) and suggests that mindset-oriented interventions may be most effective when tuned to disciplinary context, rather than applied uniformly across student groups (Anderson et al., 2018; Boaler et al., 2021; Dweck, 2006; Yeager and Dweck, 2012).

Taken together, the findings indicate that disciplinary context serves as a prominent organizing factor for mathematical mindset patterns within this sample, whereas gender-related differences appear comparatively limited and context-dependent. Rather than suggesting uniform effects across students, these results highlight the importance of examining mindset development within specific academic fields, where institutional expectations, curricular emphases, and learning practices may differentially shape students’ beliefs and motivational orientations toward mathematics.

### 5.2 From trends to theory: rethinking mathematical mindset

While the intervention yielded only modest group-level improvements in mindset scores, disaggregated analyses revealed clear and theoretically meaningful pattern: academic majors emerged as a more salient source of variation in mathematical mindset than gender when mindset was examined across multiple dimensions. Students in Engineering and Technology exhibited more favorable profiles—characterized by higher scores in growth-oriented dimensions such as Growth Mindset (GM), Learning Methods and Collaboration (LMS), and Problem-Solving Strategies (PLS)—compared to their peers in Art and Business (see Figures 5, 6). These patterns are consistent with the view that disciplinary context is closely associated with students’ motivational orientations toward mathematics, with STEM programs typically positioning mathematics as a central component of academic practice, while non-STEM fields may provide fewer sustained opportunities for mathematical engagement. These discipline-based differences were evident at both pre- and post-intervention time points, suggesting that academic major represents an important source of variation in students’ mathematical mindsets. Engineering students, in particular, showed higher scores across all five measured dimensions (GM, ML, LMS, PLS, and TBS), with especially pronounced strengths in GM and PLS, indicating comparatively stronger motivational beliefs and strategic learning tendencies that remained stable over time.

Although these discipline-based patterns highlight the stability of mindset differences across academic contexts, they do not preclude variation in how specific mindset dimensions respond to intervention. The individualized change trajectories (Figure 6) suggest that some dimensions of mindset—particularly Growth Mindset and Technology and Basic Skills—are more responsive to intervention than others. This aligns with the theoretical framework proposed by Boaler and colleagues in YouCubed, which defines mathematical mindset across five interrelated dimensions: (1) growth mindset culture (e.g., praising effort, mindset messages), (2) nature of mathematics (e.g., open tasks, depth over speed), (3) challenges and struggles (e.g., learning from mistakes, persistence), (4) connections and collaboration (e.g., peer interaction, whole-class discussion), and (5) assessment (e.g., formative feedback, low-stakes testing) (Boaler, 2016; Boaler & Williams, n.d.). Our observed post-intervention gains in LMS and TBS, particularly among Engineering and Technology students, resonate with the “connections and collaboration” and “assessment” domains emphasized by YouCubed. Conversely, the limited improvement in students’ self-reported problem-solving ability, as captured by the Mathematical Problem-Solving (PLS) scale, may reflect the relative difficulty of shifting more deeply rooted epistemological beliefs about mathematics, such as viewing mathematics primarily as the application of fixed procedures—a challenge noted in prior research (Schoenfeld, 1988; Sisk et al., 2018).

Further complicating the narrative is the weak and gendered relationship between mindset and achievement (Figure 3). While a positive association was observed between mathematical mindset and post-test performance (POMA), this link was minimal and more evident among male students. The gendered slope differences suggest that mindset-performance mechanisms may be contingent on motivational or contextual moderators, such as classroom dynamics, instructional language, or historical identity in mathematics (Eccles and Wigfield, 2002). This asymmetry suggests that interventions may be more effective when targeting malleable, skill-adjacent constructs (e.g., error tolerance, collaboration, or technical self-efficacy) rather than deeply entrenched epistemological orientations (e.g., viewing mathematics primarily as the application of fixed procedures to obtain correct answers).

Another layer of heterogeneity in our findings concerns baseline student ability. The overall similarity between control and intervention groups suggests that interventions may exert limited additional benefits for students who already exhibit strong motivational orientations and self-efficacy. By contrast, students starting with weaker belief structures or lower confidence appear more likely to show measurable gains, particularly in mindset dimensions such as tolerance for mistakes or collaborative learning. This aligns with prior work indicating that mindset interventions are most effective among students at risk of disengagement (Yeager et al., 2019), while high-achieving students often display ceiling effects. Our results therefore suggest that mindset malleability is not only discipline-specific but also stratified by students’ initial academic and motivational profiles.

Overall, these patterns point to the importance of a nuanced, dimension-specific account of mathematical mindset change. Rather than assuming uniform malleability across learners, our findings underscore the importance of context—particularly disciplinary background—in shaping both the baseline structure and responsiveness of students’ mindsets. Interventions should move beyond generic growth mindset messaging and instead recognize that mathematical mindset is multidimensional. It is therefore beneficial to collect measures and design activities along specific dimensions, such as those proposed by Boaler’s YouCubed framework—for example, providing open-ended tasks that allow students to see that they can grow and learn, encouraging mistake-driven learning to embrace challenges and struggles, and creating structured peer dialogue to foster connections and collaboration. In other words, the relationship between mindset and achievement may be less causal than conditional: beliefs support performance only when reinforced by specific institutional and cultural affordances. Future research should also investigate the longitudinal interplay between mindset dimensions and academic identity, particularly in underperforming disciplines such as the arts, where belief transformation may require deeper curricular integration and affective scaffolding.

## 6 Conclusion

This study advances understanding of mathematical mindset as a multidimensional construct shaped by academic context. Although the overall intervention effects were modest at the aggregate level, disaggregated analyses revealed consistent, theoretically meaningful patterns across disciplines. Students in engineering-related fields tended to endorse more growth-oriented beliefs across multiple dimensions, whereas gender-related differences were comparatively small and context-dependent. The weak and uneven association between mindset and achievement highlights the complexity of translating beliefs into performance and cautions against assuming uniform or linear effects.

Collectively, these findings challenge simplified assumptions about mindset malleability and underscore the importance of designing interventions that are sensitive to disciplinary cultures, epistemological orientations, and students’ baseline motivational profiles. Rather than relying on generic growth mindset messaging, future interventions may benefit from a dimension-specific approach that integrates curricular design, instructional practice, and institutional context. Future research should further examine these relationships using longitudinal designs and richer contextual measures to better capture the conditional pathways through which mindset supports learning and achievement.

## 7 Limitations and future directions

While the intervention showed dimension-specific impact, its group-level effects were relatively limited. As shown in Figure 2, students in the intervention group reported higher post-intervention MMH scores, reflecting increased affective engagement and confidence, though the magnitude of change was modest. In contrast, Figure 3 demonstrates a statistically significant reduction in MML scores (*p* = 4.5e-07), particularly among students with initially rigid mindsets, suggesting that belief-based dimensions were more susceptible to short-term change. However, Figure 3 revealed consistently weak correlations between mindset (MMH and MML) and mathematics achievement (POMA), indicating that improved beliefs did not directly translate into performance gains. These findings underscore the complexity of mindset transformation: while some cognitive-affective dimensions may be malleable, they do not automatically result in measurable academic improvement. Moreover, the present trial was conducted with college students in China, embedded in a selective and exam-oriented higher-education system, so the psychological meaning of “mindset” and the classroom practices through which it is enacted may differ from those in other educational systems and age groups (Li, 2004, 2012; Walton and Wilson, 2018). Caution is therefore warranted when extrapolating the present pattern of effects to younger learners or to students in different cultural and institutional contexts.

Future studies should consider both the temporal dynamics and contextual moderators of mindset change. As our data suggest, academic major played a far more decisive role than gender in shaping both baseline profiles and post-intervention outcomes—Engineering majors consistently outperformed other disciplines across all five mindset dimensions, particularly in GM and PLS (see Figure 6). This pattern is consistent with accounts that conceptualize academic fields as distinct “motivational cultures” that organize opportunity structures, identity signals, and perceived affordances for learners (Leslie et al., 2015). Gender differences, while present, were minor and often embedded within major-specific trajectories. Therefore, future research should focus less on binary gender comparisons and more on how gender interacts with disciplinary norms, motivation, and learning environments.

In addition, cross-cultural and cross-age replications—for example, with primary and secondary students and in non-Chinese educational systems—are needed to test the robustness and boundary conditions of these findings and to clarify when and for whom mindset-oriented interventions are most effective (Henrich et al., 2010; Walton and Wilson, 2018). Finally, a longer intervention duration, the use of behavioral or performance-based measures, and inclusion of longitudinal follow-up would strengthen the causal inferences and help illuminate whether mindset shifts can lead to sustained academic transformation. At the same time, the present analyses are exploratory in nature and do not establish causal relationships between mathematical mindset, academic major, and achievement; observed differences should therefore be interpreted as contextually patterned associations rather than as evidence of direct intervention effects. Moreover, although the study was conducted within Chinese higher education, the findings are not intended to be culturally deterministic; rather, China serves as an institutional context through which the interaction between disciplinary structure, gender, and mathematical mindset can be examined, and caution is warranted when generalizing beyond similar settings.

## Data Availability

The datasets presented in this article are not readily available because the raw data supporting the conclusions of this article will be made available upon reasonable request to the corresponding author. Requests to access the datasets should be directed to sophiexiaoyuxu@163.com.

## Appendix

**Table A1:** Internal consistency estimates of the mathematical mindset subscales.

Subscale	Cronbach’s α
1. PrGM	0.797
2. PrLMS	0.487
3. PrML	0.003
4. PrPLS	0.358
5. PrTBS	0.274
6. PoGM	0.884
7. PoLMS	0.653
8. PoML	0.299
9. PoPLS	0.313
10. PoTBS	0.059

Pr = pre-test; Po = post-test. Cronbach’s α values are reported for descriptive purposes. Several subscales contain a small number of items (particularly two-item subscales), for which internal consistency estimates may be attenuated and should be interpreted with caution. Given the exploratory nature of the study and the conceptually heterogeneous structure of mathematical mindset, reliability coefficients were not used as the primary basis for interpretation.
